# Cost-effectiveness of the Australian Medical Sheepskin for the prevention of pressure ulcers in somatic nursing home patients: study protocol for a prospective multi-centre randomised controlled trial (ISRCTN17553857)

**DOI:** 10.1186/1472-6963-8-4

**Published:** 2008-01-07

**Authors:** Patriek Mistiaen, Wilco Achterberg, Andre Ament, Ruud Halfens, Janneke Huizinga, Ken Montgomery, Henri Post, Anneke L Francke

**Affiliations:** 1 NIVEL, Netherlands Institute for Health Services Research, PO Box 1568, 3500 BN Utrecht, The Netherlands; 2 VU University Medical Center, EMGO institute, Van de Boechorststraat 7, 1081 ET Amsterdam, The Netherlands; 3 University of Maastricht, PO Box 616, 6200 MD Maastricht, The Netherlands; 4 V&VN-Dermatology, National Nursing and Caring Organisation, department for dermatology nursing, PO Box 8212, 3503 RE Utrecht, The Netherlands; 5 Ken Montgomery Consulting, Milan street 4/unit 37, VIC 3194 Mentone, Australia; 6 Evean Zorg, Bristolroodstraat 164, 1503 NZ Zaandam, The Netherlands

## Abstract

**Background:**

Pressure ulcers are a major problem, especially in nursing home patients, although they are regarded as preventable and there are many pressure relieving methods and materials. One such pressure relieving material is the recently developed Australian Medical Sheepskin, which has been shown in two randomized controlled trials [1,2] to be an effective intervention in the prevention of sacral pressure ulcers in hospital patients. However, the use of sheepskins has been debated and in general discouraged by most pressure ulcer working groups and pressure ulcer guidelines, but these debates were based on old forms of sheepskins. Furthermore, nothing is yet known about the (cost-)effectiveness of the Australian Medical sheepskin in nursing home patients.

The objective of this study is to assess the effects and costs of the use of the Australian Medical Sheepskin combined with usual care with regard to the prevention of sacral pressure ulcers in somatic nursing home patients, versus usual care only.

**Methods/Design:**

In a multi-centre randomised controlled trial 750 patients admitted for a primarily somatic reason to one of the five participating nursing homes, and not having pressure ulcers on the sacrum at admission, will be randomized to either usual care only or usual care plus the use of the Australian Medical Sheepskin as an overlay on the mattress.

Outcome measures are: incidence of sacral pressure ulcers in the first month after admission; sacrum pressure ulcer free days; costs; patient comfort; and ease of use. The skin of all the patients will be observed once a day from admission on for 30 days. Patient characteristics and pressure risk scores are assessed at admission and at day 30 after it.

Additional to the empirical phase, systematic reviews will be performed in order to obtain data for economic weighting and modelling.

The protocol is registered in the Controlled Trial Register as ISRCTN17553857.

## Background

"*How to prevent or heal pressure sores is an ever-present problem for nurses, and they have had many and varied ideas for solving it. Some of their methods have been helpful; others have been failures. Sometimes old methods that were not 100 per cent effective have been put aside for new ones, and, occasionally, an old one is revived to be tried again. Such a method is sheepskin therapy (Woodruff, **1952**) *[3]."

This historical quote is interesting, since it shows that pressure ulcers have been a major problem for many years. Furthermore it is interesting because it states that old therapies sometimes are revived, such as the sheepskin. More than 50 years later the sheepskin, now labelled as the 'Australian Medical Sheepskin', has its second revival.

Pressure ulcers are highly prevalent, especially among elderly residing in nursing homes, where about 30% of the patients are suffering from these sores [4-7]; a Canadian study in two nursing homes even found a prevalence rate up to 53% [8]. A USA-study in 12 nursing homes found a mean incidence rate of 13.6%, varying between 3 and 31% [9]. Incidence rates for Dutch nursing homes are scarce: one study found an incidence rate of 25% in the first four weeks after admission [10]. Pressure ulcers are not just a very common problem, they also represent a major burden and reduce quality of life for patients and their carers; pressure ulcers cause prolonged contact with the health care system, pain, discomfort and inconvenience [11-15].

Pressure ulcers are considered as a problem that to a large extent can be prevented by good care; therefore the incidence and prevalence rates are now put forward by many health care, professional and governmental organizations as an indicator of the quality of health care [16-20].

The prevention and therapy of pressure ulcers are very costly [13,21-25]. However, economic evaluations of pressure-relieving devices for the prevention of pressure ulcers are few and far between [26].

Pressure ulcers develop when capillary circulation is obstructed by prolonged pressure. Also shear and friction are contributing factors for the development of pressure ulcers. Therefore many interventions are in use to minimize pressure on bony prominences, such as repositioning and different kinds of pressure relieving devices [23,27,28]. Sheepskin is one such pressure relieving device. According to a literature review of the Australian Commonwealth Scientific and Industrial Research Organization (CSIRO) [29], it is known that sheepskins have pressure relieving properties, as was already demonstrated in studies dating back to the 1930s, 1950s and 1960s. Besides this, sheepskins have good moisture absorbing capacities. Therefore sheepskins have long been in use to prevent pressure ulcers. However, the old natural sheepskins could not retain their pressure reducing capacities after frequent washings; hygienic problems also arose because they could not withstand washing above 40 degrees. Later many attempts were made to produce artificial sheepskins, but none had the same pressure reducing or moisture absorbing properties. And so the use of sheepskins, both genuine and artificial, was almost completely discontinued and came to be regarded as ineffective [30]. Currently none of the guidelines known to us on pressure ulcer prevention and treatment mention or encourage the application of any form of sheepskin. However, in the 1990s, interest in genuine sheepskin was renewed. Marchand *et al.*[31] found promising results, which prompted the Australian Commonwealth Scientific and Industrial Research Organization, CSIRO, to develop new methods for producing new genuine medical sheepskins with very high pressure reducing capacities but without the disadvantages of the old sheepskins. The Australian Medical Sheepskin is a real genuine sheepskin, coming from the Australian Merino sheep. The sheepskin has a wool pile length of 30 mm, and a high density of wool piles. The sheepskin is tanned and processed in such a way that it has an increased resistance to urine and can withstand up to 60 washes at 80°C to achieve high-level thermal disinfection, without losing its moisture absorbing and pressure relieving properties.

This new Australian Medical Sheepskin has been tested extensively on pressure relieving characteristics, laundry procedures, disinfection, wear-and-tear, patient comfort and other aspects [32]. The necessary qualities for a sheepskin to be called an Australian Medical Sheepskin are described in the National Australian Standard AS 4480.1 [33]. The effectiveness of the Australian Medical Sheepskin in the prevention of pressure ulcers was first tested clinically in a randomized controlled trial in 297 elderly orthopedic hospitalized patients with very good results: incidence rate of pressure ulcers was 9.6% in the intervention versus 30% in the control group [1]. These results were confirmed in another randomized trial with 441 adults patients from different hospital wards [2]. Both studies were well designed, with the latter awarded for the best research article published in the Medical Journal of Australia of 2004. The application of Australian Medical Sheepskins is very easy for nursing and care personnel and for patients [1,2]. These trial results led to positive comments and recommendations in three recent systematic reviews [27,28,34].

However, the question arises as to whether these results in acute hospital patients can be replicated in the more chronic and vulnerable nursing home patients, who are generally at higher risk for developing pressure ulcers. In addition, in the Australian studies the cost-effectiveness was not assessed.

We therefore consider it is worthwhile to study the effects in nursing home patients and to assess the cost-effectiveness of the Australian Medical Sheepskin.

## Methods/Design

### Research aims and questions

The purpose of this study is to assess the costs and effects of the Australian Medical Sheepskin combined with usual care in the prevention of sacral pressure ulcers in nursing home patients, versus usual care only.

Research questions are:

1. What are the effects of the application of Australian Medical Sheepskins on the incidence rates of sacral pressure ulcers in somatic nursing home patients in the first month after admission compared to usual care?

2. What are the costs of the application of the Australian Medical Sheepskin?

3. What are the costs of the treatment for eventually developed pressure ulcers in the two study groups?

4. What is the cost-effectiveness of the Australian Medical Sheepskin compared to usual care?

5. What adverse effects do Australian Medical Sheepskins have?

6. How do patients and nursing and care personnel rate the (dis)comfort of Australian Medical Sheepskins and the ease of use of Australian Medical Sheepskins?

### Design

The proposed study concerns a prospective multi-centre randomised controlled trial with one experimental and one control group. The starting point is a superiority design with the research hypothesis that Australian Medical Sheepskins are a better pressure relieving device than usual care; that they will reduce the incidence rates of sacral pressure ulcers; and will also reduce costs involving pressure ulcer treatment.

Data will be gathered primarily in an empirical way and additionally through systematic literature searches with regard to the cost-effectiveness question.

For assessing the cost-effectiveness, the Australian Medical Sheepskin will be compared only to usual care and not to other pressure relieving materials. The perspective of the health care organisation will be considered to measure the cost-effectiveness.

### Participants

The study population will concern patients newly admitted for a primarily somatic condition to one of the five participating nursing homes. Only patients who are free of sacral pressure ulcers on admission will be included. Further inclusion criteria are: adult; not having darkly pigmented skin (because of difficulty in diagnosing grade 1 pressure ulcer); having an expected stay of more than one week; and consenting to the research.

For practical reasons and because 74% of nursing home patients in the Netherlands are at risk for pressure ulcer development [4], we have chosen to involve all patients, and not only patients at risk.

The patients will be recruited from 5 different Dutch nursing home care groups (Amstelring, Florence, Frankeland, Sutfene, Swinhove) involving seven different geographical locations and 18 wards. The selection of the nursing homes was made on the basis of the customer list of the laundry company that we contacted, and which is able to do the washing and drying of the sheepskin, according to the Australian Standard [35].

Inclusion of patients started on May 1^st ^2007 and will proceed until the targeted number of patients is reached, for a maximum of 1.5 years. It is expected that enough patients will be admitted during this period to reach the necessary number as was calculated by power-analysis.

### Sample size calculation

In Dutch nursing homes the prevalence of pressure ulcers is about 30% for all grades and about 12% for grade 2 to 4 [4]. Few studies have investigated incidence rates for Dutch nursing homes: one study found an incidence rate of 25% in the first four weeks after admission [10]. The first studies [1,2] on the Australian Medical Sheepskin found incidence rates of 30% in the control group and 9.6% for the intervention group.

For this study we will consider the Australian Medical Sheepskin to be effective and clinically significant if the incidence rate in the first month after admission is at least (in absolute terms) 10 percentage points lower in the experimental group than in the control group. This means that with an expected maximum incidence rate of 18% in the experimental group and about 28% in the control group, 750 patients (2 × 375) are needed to find such differences with a power of at least 80% and a two-sided alpha of 0.05.

**Figure 1:**
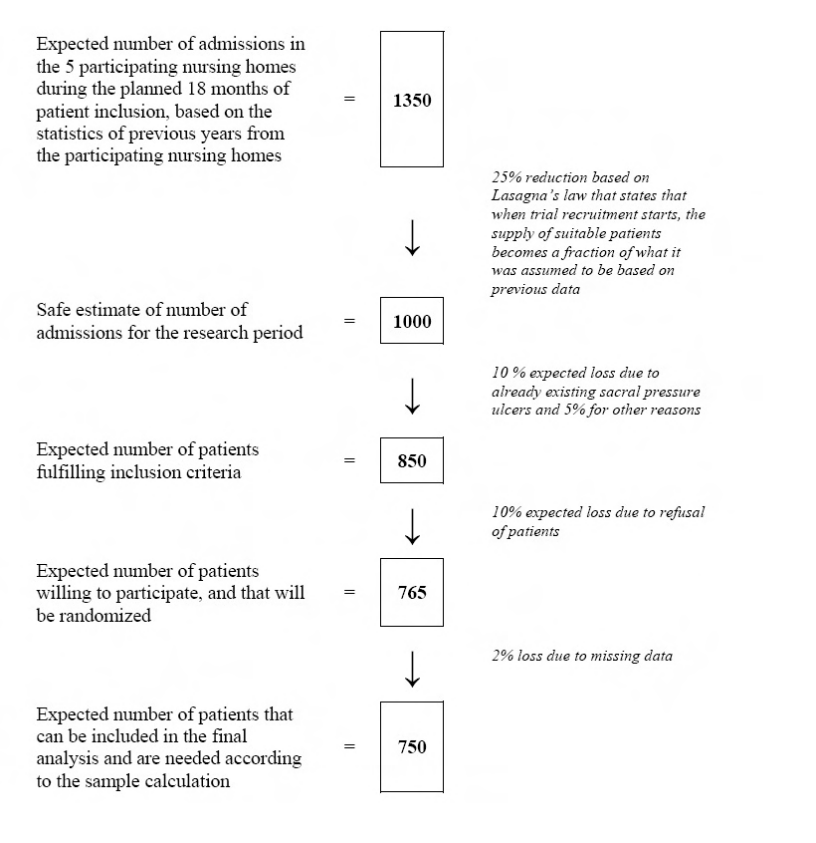
Flow chart expected patient inclusion.

Halfens *et al.*[4] state that 68% of the pressure ulcers in nursing homes originate during the nursing home stay; accordingly, with a prevalence of 30%, this means that approximately 10% of the patients are admitted with an already existing pressure ulcer, and will therefore not fulfill our inclusion criteria. Furthermore, it is expected that about 10% of the patients will refuse to participate and that about 5% will drop-out from the study for other reasons (e.g. not being able to discuss matters and give informed consent). Allowing for drop-out and for the fact that not all patients are pressure ulcer free when admitted to the nursing home, 1000 patients (2 × 500) will be recruited. Based on the admission data of previous years, it is assumed that about 1,350 patients will be admitted in the planned 18 months of patient inclusion. The expected patient inclusion flow is shown in Figure 1.

### Patient allocation/randomisation sequence/blinding

All newly admitted patients will be checked by the nurse in charge at admission to ascertain whether they fit the inclusion criteria. If so, they will be given research information in written format and verbally by the same nurse at admission. On the next day they will be asked for informed consent (by the same nurse) and if they agree, the nurse will immediately call the central research office to find out to which group the patient has been randomized. Thus, the therapy according to randomization status will start immediately and within less than 48 hours after admission.

To ensure concealment of allocation, a randomization scheme was created in SPSS by assigning the intervention to a random sample of circa 50% in a list of 1,500 numbers and assigning the control group to the rest. This allocation of the group (sheepskin, usual care) was then blinded on a paper list numbered 1 through 1,500 by a secretary not further involved in the project. Each time a patient is admitted, the principal investigator located at the central research office is called. The above list with numbers and blinded allocation is held at this office. This central research office registers each patient identity for every consecutive number and discloses the group allocation to the nurse and patient.

Only the patient allocation itself is blinded to all parties involved, because it is impossible to blind health professionals or patients to whether someone is in the sheepskin group or not.

### Intervention

The experimental condition consists of the application of the Australian Medical Sheepskin as an overlay on top of the standard/usual mattress in the area of the buttocks and in the (wheel)chair, combined with all care as usual. An extra Australian Medical Sheepskin at the bottom of the bed is also permitted. The intervention will start no later than 48 hours after admission. The Australian Medical Sheepskin will be applied during the first 30 days after admission (and prolonged if the patients wish so, but without gathering research data). The period of 30 days is chosen because most pressure ulcers develop in the first weeks after admission [6,10,36,37]. The sheepskins will be changed at least every two days.

Besides the use of the Australian Medical Sheepskin, all other usual pressure ulcer preventative interventions such as mobilization and repositioning may be added as co-interventions, in so far as they are normally used in the participating nursing homes. All other nursing care can be continued as usual (including incontinence materials).

The control group will receive usual care, including all the pressure relieving interventions and other preventive actions, normally taken in the participating nursing homes. The application, in any way, of the Australian Medical Sheepskin is forbidden in this group.

### Outcomes

A distinction can be made between variables relating to the effectiveness (incidence, prevalence of pressure ulcers) and variables relating to costs (purchase and washing of the sheepskins on the one hand, and investment of time and materials cost for pressure ulcer treatment on the other). Both of these are necessary to compute cost-effectiveness.

The primary outcomes for this study are:

- the incidence of sacral pressure ulcers in the first 30 days after admissions,

- the costs of appliance of the sheepskin, and

- the costs related to treatment of the pressure ulcers that have developed.

Secondary outcomes are the presence of pressure ulcers on other places, quality of life, comfort of the sheepskin as experienced by the patients and ease of use of the sheepskin as experienced by the care personnel.

Influencing factors that will be measured are patient characteristics, the application of other pressure relieving interventions and materials and the use of incontinence materials.

### Instruments

These outcomes, as well as influencing factors, will be measured by appropriate instruments, as far as possible we will use already existing and validated instruments. The EPUAP grading system is used to assess the presence and severity of pressure ulcers [38].

To this end a 'daily observation checklist' has been developed. Nurses use this checklist during the first 30 days after admission to report the presence/absence of sacral pressure ulcers, as well as the ulcer grade; the presence/absence of pressure ulcers on other locations and whether a sheepskin, repositioning, other support surfaces and incontinence material are applied.

When nurses feel uncertain about their observations they are instructed to call another specialized nurse, experienced in observing pressure ulcers.

Furthermore, a photographic series of the various pressure ulcer grades is available on each ward, as well as special glasses that can be used in order to differentiate better between blanchable and non-blanchable erythema. Finally, all nursing homes are offered a lesson in pressure ulcer observation.

The daily skin observations by nurses during routine care will be checked weekly by an experienced nurse observator from the nursing home itself and bimonthly by the principal investigator from the central research office.

Influencing patient factors that will be measured are age, gender, principal medical diagnosis category, surgery in the last month, presence of pressure ulcers at admission on other places than the sacrum, incontinence status, nutritional status (including body mass index), activity of daily living level (Barthel-index) and pressure ulcer risk score (Braden-scale). These are measured twice: at admission and at day 30 after it.

Furthermore the patients in the sheepskin group are asked at the end of the research period to rate the (dis)comfort of the sheepskin. For this purpose, a self-developed 7-item questionnaire with a 5-point rating answer structure was developed.

Data about costs that will be gathered are the costs of buying sheepskins, costs of washing sheepskins, costs of treatment of pressure ulcers (medication, wound care materials, diagnostics, kind of specialized consultants and time involved, and extra time for nurses/carers with regard to pressure ulcer treatment).

For this purpose, invoices from the firms in question will be used, or the best approximations as far as these are available in Dutch (governmental) sites/organizations.

In summary, in order to register the above-mentioned data, the following five forms were created: admission form, daily observation form, end of research period form, sheepskin comfort questionnaire and the pressure ulcer therapy cost form (these forms in Dutch can be obtained on request from the first author).

The initial assessment at admission is completed for all patients that fulfill the inclusion criteria, in order to conduct a post hoc check as to whether there was selective attrition in patients not willing to participate.

Ease of use of the sheepskin for carers will be measured by group interviews to be held on three occasions during the research period.

### Data-analysis

First of all the primary outcomes will be analyzed using descriptive methods.

Statistical testing of differences in incidence and prevalence between experimental and control groups will be conducted by a two-sided Chi-square test. In addition, differences in number of pressure ulcer free days will be tested with a two-sided t-test for independent samples; and differences in onset of pressure ulcers will be analyzed with Kaplan Meier curves. All analyses will be controlled for influencing factors. Moreover, a multi level analysis will be performed because of the fact that data are gathered from several organizations and nursing wards.

Both intention-to-treat-analysis and per-protocol-analysis will be carried out.

The economic data about costs will be used in a model approach to show the economic consequences on a higher level. The aim is to develop a model that can show the consequences of implementing improved pressure ulcer care for an average nursing home and/or the national level.

### Ethical considerations

Patients will be informed verbally and in written format about the research project before they are asked to participate and before they sign the informed consent form, which will also be signed by the nurse in charge at admission. At all times patients are free to stop their participation without having to give a reason for doing so.

The study protocol has been reviewed and approved by the certified Medical Ethical Review Board of the University Medical Centre of Utrecht (protocol number 06/287), on behalf of all the participating nursing homes.

The Board of directors of each participating nursing home signed a form in which they state they have read and understood the research protocol and that there are no obstacles at all to perform the research in their organizations.

The scientific merits of the study protocol have been reviewed in the consecutive phases of research funding process by the independent reviewers of the funding organization ZonMw, the Netherlands Organization for Health Research and Development [39]. The ZonMw grant obtained is number 945-07-513.

Next to this, the research protocol, and more specifically the washing procedure of the sheepskins, has also been judged by the Dutch Working Party on Infection Prevention [40] and has been approved as safe.

### Additional systematic reviews

In addition to the empirical data gathering, systematic literature reviews will be performed to retrieve adjuvant data concerning:

- costs of pressure ulcer treatment,

- pressure ulcer incidence and duration per grade in nursing home patients, and

- influence of pressure ulcers on quality of life.

These reviews will be conducted in order to compare the empirically found cost-effectiveness data to other studies, and to improve economic modelling and scenarios.

For these systematic reviews a protocol has been written including databases to be searched, search strategies, inclusion criteria and process, methodological assessment and data-analysis. These review protocols (in Dutch) can be obtained from the authors.

### Publication

All results from this study will be submitted to suitable peer reviewed journals and will be presented at appropriate international conferences.

The patient inclusion process started on May 1^st ^2007 and is expected to continue until the end of 2008. First results are not expected earlier than mid-2009.

### Funding

A grant (number 945-07-513) was obtained in a competitive application process of the efficacy research program, round 2007, of the Netherlands Organization for Health Research and Development ZonMw [39].

## Discussion

This study is innovative because it is the first to assess the cost-effectiveness of the Australian Medical Sheepskin in nursing home patients and also because current opinions and guidelines [13,41-44] oppose the use of sheepskins. However, these are based on old forms of sheepskins, not comparable to the genuine Australian Medical Sheepskin.

A difficult point in this research is that the usual care can not be standardized. Although nurses and nursing homes may have pressure ulcer protocols and say that they are following these, there is a chance that the usual care differs from nursing home to nursing home, or even from nursing ward to nursing ward, and that individual nurses may deviate from the protocol. Moreover, it is known that different mattresses are in use in and between nursing homes. Since standardization is in no way possible, a registration of mattresses and devices applied will be conducted.

With regard to possible contamination between experimental and control group, the following measures will be taken: clear instruction to all nursing and care personnel and patients, a limited stock of sheepskin on the ward, daily inspection of the skin by the research team in both groups, and a concise control and observation to ascertain whether the patients are still being nursed in the condition they were randomized to. Moreover it is not very likely that patients from both groups will be nursed on the same room during the same period (1.2 admissions per bed); therefore it is improbable that patients from the control group will ask for a sheepskin.

Finally, in the information sessions with nurses, it will be stressed that the Australian Medical Sheepskin is NOT a replacement for the normal pressure relieving interventions such as repositioning and other methods.

With regard to treatment adherence, patients from the intervention group are asked only to keep the sheepskin in their bed for 30 days. No other action is required from patients other than actions that are also asked in the control group. However it remains possible that some patients after a few days will refuse the sheepskin (e.g. because of itching, not feeling comfortable, too hot, or other reason): in these cases the use of the Australian Medical Sheepskin will be stopped without persuading patients to continue, but they will be asked if the daily skin observations can be continued. Records will be kept of patients that have discontinued the use of the Australian Medical Sheepskin (including reasons to stop), and they will be analyzed in the group they were randomized, according to intention to treat analysis principles.

## Conclusion

The Australian Medical Sheepskin is a new pressure relieving overlay, which has been proven to be effective to prevent pressure ulcers in two randomized controlled trials among hospital patients. However, it is not known yet if the Australian Medical Sheepskin is also an effective prevention for pressure ulcers in nursing home patients; nor is it known what the cost-effectiveness characteristics are of the Australian Medical Sheepskin are. This study will attempt to answer these questions.

## Competing interests

The author(s) declare that they have no competing interests.

The sheepskins were purchased, after having requested trade offers from three different companies, at regular market prices from Yellow Earth, Laverton North, Australia.

The washing procedures of the sheepskins are carried out at regular market prices by Lips Textile Services, Tiel, The Netherlands.

These two firms do not contribute in any way to the funding of this study, and none of the authors have commercial or financial interests with these companies.

## Authors' contributions

All authors contributed substantially to the development of the study protocol and the drafting of this paper. All authors read and approved the final manuscript.

## Pre-publication history

The pre-publication history for this paper can be accessed here:


